# Inflammation and mitophagy are mitochondrial checkpoints to aging

**DOI:** 10.1038/s41467-024-47840-1

**Published:** 2024-04-20

**Authors:** Emma Guilbaud, Kristopher A. Sarosiek, Lorenzo Galluzzi

**Affiliations:** 1 Department of Radiation Oncology, Weill Cornell Medicine, New York, NY USA; 2 John B. Little Center for Radiation Sciences, Harvard T.H. Chan School of Public Health, Boston, MA USA; 3 Sandra and Edward Meyer Cancer Center, New York, NY USA; 4 Caryl and Israel Englander Institute for Precision Medicine, New York, NY USA

**Keywords:** Mitochondria, Ageing

## Abstract

Cellular and organismal aging have been consistently associated with mitochondrial dysfunction and inflammation. Accumulating evidence indicates that aging-related inflammatory responses are mechanistically linked to compromised mitochondrial integrity coupled with mtDNA-driven CGAS activation, a process that is tonically inhibited by mitophagy.

Both cellular senescence and organismal aging are accompanied by a multitude of pathophysiological alterations, encompassing (but not limited to) accumulating oxidative damage to macromolecules including DNA, impaired metabolism, and inflammation^1^. All these defects are intimately connected with yet another hallmark of aging: mitochondrial dysfunction^1^. Indeed, defective mitochondria are prone to overproduce reactive oxygen species (ROS), are unable to support physiological metabolism, and can elicit potent inflammatory reactions^1,2^. Importantly, aging biological systems also exhibit a decrease in the proficiency of homeostatic processes such as autophagy, a lysosome-dependent mechanism for the degradation of damaged or otherwise potentially harmful cytoplasmic entities^1^. In line with this notion, multiple experimental maneuvers that promote autophagy (such as caloric restriction) have been shown to extend lifespan in a variety of model organisms including mice^1^.

That said, whether aging-associated alterations emerge as a consequence of defects in cellular adaptation or instead perturbations of homeostasis ultimately overwhelm the cellular capacity for adaptation remains to be formally elucidated. Irrespective of this and other unknown, the acquisition of a senescent phenotype by aging cells has been shown to involve an autocrine/paracrine mechanism linked to type I interferon (IFN) signaling as elicited not only by DNA damage^3^, but also by mitochondrial dysfunction^4^. Thus, mitochondrial integrity stands out as a major gatekeeper for the control of genetic, metabolic, and inflammatory homeostasis^1^. Recent data from Jimenez-Loygorri et al. demonstrate that mitophagy (a specialized variant autophagy that degrades defective mitochondria)^5^ limits aging-associating neurological decline by suppressing inflammatory reactions driven by primary mitochondrial dysfunction coupled with mitochondrial DNA (mtDNA) release and consequent cyclic GMP-AMP synthase (CGAS) signaling^6^.

Jimenez-Loygorri et al. set out to examine the activation of the molecular machinery for mitophagy across different organs in old *versus* young mice expressing a pH-sensitive reporter that enables the discrimination of cytosolic (mCherry^+^GFP^+^) *versus* lysosomal (mCherry^+^GFP^−^) mitochondria (so-called mito-QC mice). Surprisingly, in some organs such as the retina, mitophagy was higher in old vs young mice. In line with this notion, the retina of aged mice exhibited increased markers of mitophagy (but not general autophagy) activation including the PTEN-induced kinase 1 (PINK1)-dependent phosphorylation of ubiquitin at S65. Such an increase in mitophagy was accompanied not only by ultrastructural markers of mitochondrial damage (*e.g*., swollen mitochondria, mitochondria exhibiting *cristae* disruption) but also by the cytosolic accumulation of mtDNA and consequent CGAS activation, culminating in (1) the stimulator of interferon response cGAMP interactor 1 (STING1)-dependent activating phosphorylation of the transcription factor interferon regulatory factor 3 (IRF3) at S396, and (2) the expression of multiple IRF3 targets, including several interferon-stimulated genes (ISGs) like interferon beta 1 (*Ifnb1*). Similar observations were obtained with primary normal human dermal fibroblasts (NHDFs) from aged individuals, globally suggesting that PINK1-mediated mitophagy is activated during aging in response to primary mitochondrial dysfunction^6^.

Next, Jimenez-Loygorri and colleagues investigated the impact of experimental interventions that alter mitophagic activity on the aging retina. Urolithin A (UA), which indirectly inhibits mechanistic target of rapamycin (MTOR) signaling, promoted mitophagy in the retina of both young and aged mice, a precess that in the latter setting was associated with significant improvements in recognition memory, night vision, synaptic integrity and limited aberrant integration of light stimuli compared to untreated old mice. Moreover, UA decreased the amount of total and CGAS-bound mtDNA in the cytosolic fraction of the retina from aged mice, which was accompanied by a reduction in genetic signatures of CGAS signaling and type I interferon (IFN) responses^6^. In line with this finding, cytosolic mtDNA accumulation as elicited by ABT-737 – which promotes mitochondrial outer membrane permeabilization (MOMP) by enabling BCL2 associated X, apoptosis regulator (BAX) and BCL2 antagonist/killer 1 (BAK1) oligomerization^7^ – and QVD – which blocks caspases to prevent the suppression of CGAS signaling downstream of MOMP^8,9^ – in immortalized retinal pigmented epithelial ARPE-19 cells was significantly inhibited by UA. Accordingly, UA not only abolished CGAS activation but also limited ROS production and restored oxidative phosphorylation in ARPE-19 cells exposed to ABT-737 and QVD. Of note, the CGAS inhibitor G140 also limited mitophagy as driven by MOMP in ARPE-19 cells, but failed to alter cytosolic mtDNA accumulation^6^. These findings suggest that mitophagic responses promoted by mitochondrial dysfunction in the aged retina may depend, at least in part, on CGAS signaling.

To obtain additional insights into their observations, Jimenez-Loygorri and collaborators co-silenced *PINK1* and parkin RBR E3 ubiquitin-protein ligase (*PRKN*, which encodes another molecular component of the mitophagy apparatus)^5^ in ARPE-19 cells exposed to ABT-737 and QVD, finding that cytosolic mtDNA accumulation as elicited by MOMP in the context of caspase inhibition is aggravated by mitophagy defects, which also abolish the effects of UA. Conversely, inhibition of mitochondrial biogenesis with chloramphenicol reduced cytosolic mtDNA accumulation as driven by ABT-737 and QVD in ARPE-19 cells while preserving their sensitivity to UA^6^.

In conclusion, Jimenez-Loygorri and colleagues demonstrated that mitophagy decelerates aging by suppressing inflammatory responses downstream of primary mitochondrial dysfunction and consequent mtDNA-dependent CGAS signaling (Fig. 1). Together with recent data from us and others^4,10–13^, these findings point to a central role for the mitochondrial checkpoint in a multitude of pathophysiological settings, including aging, autoimmunity, adaptive immune responses as well as cancer sensitivity to (immuno)therapeutics. Thus, since mitophagy acts as a major gatekeeper of the mitochondrial checkpoint, pharmacological strategies to enforce it (mitophagy activators) or weaken it (mitophagy inhibitors) may have broad therapeutic applications. Importantly, the activation of apoptotic executioner caspases as elicited by widespread MOMP has been consistently shown to suppress CGAS signaling by a variety of mechanisms^14,15^. In line with this notion, aging cells appear to experience sublethal degrees of MOMP (also known as minority MOMP) that are compatible with cell survival but promote senescence and inflammatory responses^2,4^. These observations raise the intriguing possibility that strategies to elicit the sublethal activation of executioner caspases in the absence of accrued MOMP might suppress aging-associated inflammation without causing cell death, as would inhibitors of CGAS, STING1, or IRF3. Additional work is required to formally investigate these possibilities.

**Fig. 1:**
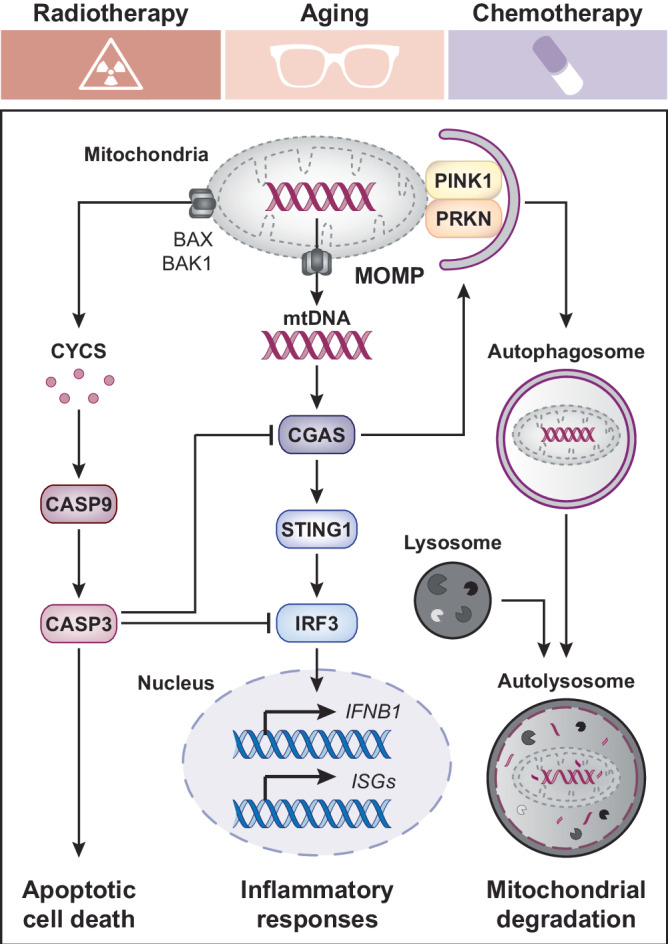
Mitophagy-dependent enforcement of the mitochondrial checkpoint in stressed and aging cells. Mitochondrial dysfunction as spontaneously emerging in aging cells or as elicited by exogenous stressors such as radiation therapy and chemotherapy can be accompanied by the permeabilization of mitochondrial membranes, hence compromising the integrity of the mitochondrial checkpoint. In this context, mitochondrial DNA (mtDNA) released or bulging from permeabilized mitochondria operates as a potent activator of cyclic GMP-AMP synthase (CGAS), hence initiating a stimulator of interferon response cGAMP interactor 1 (STING1)-dependent signaling cascade that culminates with the transactivation of multiple interferon-stimulated genes (ISGs), including interferon beta 1 (*IFNB1*). The efficient removal of compromised mitochondria as ensured by PTEN induced kinase 1 (PINK1)- and parkin RBR E3 ubiquitin protein ligase (PRKN)-dependent mitophagy tonically suppresses such an aging- and stress-associated inflammatory phenotype. Of note, mitochondrial outer membrane permeabilization (MOMP) as mediated by BCL2 associated X, apoptosis regulator (BAX) and BCL2 antagonist/killer 1 (BAK1) is also associated with the release of cytochrome c, somatic (CYCS), culminating in at least some degree of caspase 9 (CASP9) and CASP3 activation, which precipitates apoptotic cell death as it suppresses CGAS signaling. Whether activating apoptotic caspases to sublethal degrees in the absence of accrued MOMP may decelerate aging by limiting mtDNA-driven CGAS activation remains to be elucidated. IRF3 interferon regulatory factor 3.
